# Safety evaluation of β-nicotinamide mononucleotide oral administration in healthy adult men and women

**DOI:** 10.1038/s41598-022-18272-y

**Published:** 2022-08-24

**Authors:** Yuichiro Fukamizu, Yoshiaki Uchida, Akari Shigekawa, Toshiya Sato, Hisayuki Kosaka, Takanobu Sakurai

**Affiliations:** 1 Research and Development Division, Mitsubishi Corporation Life Sciences Limited, 1-1-3 Yurakucho, Chiyoda-ku, Tokyo, 100-0006 Japan; 2 Takaishi Fujii Hospital, 1-14-25 Ayazono, Takaishi-shi, Ōsaka, 592-0014 Japan

**Keywords:** Health care, Medical research

## Abstract

A decrease in the intracellular level of nicotinamide adenine dinucleotide (NAD+), an essential coenzyme for metabolic activity, causes various age-related diseases and metabolic abnormalities. Both *in-vivo* and *in-vitro* studies have shown that increasing certain NAD+ levels in cell or tissue by supplementing nicotinamide mononucleotide (NMN), a precursor of NAD+, alleviates age-related diseases and metabolic disorders. In recent years, several clinical trials have been performed to elucidate NMN efficacy in humans. However, previous clinical studies with NMN have not reported on the safety of repeated daily oral administration of ≥ 1000 mg/shot in healthy adult men and women, and human clinical trials on NMN safety are limited. Therefore, we conducted a randomized, double-blind, placebo-controlled, parallel-group study to evaluate the safety of 1250 mg of β-NMN administered orally once daily for up to 4 weeks in 31 healthy adult men and women aged 20–65 years. Oral administration of β-NMN did not result in changes exceeding physiological variations in multiple clinical trials, including anthropometry, hematological, biochemical, urine, and body composition analyses. Moreover, no severe adverse events were observed during the study period. Our results indicate that β-NMN is safe and well-tolerated in healthy adult men and women an oral dose of 1250 mg once daily for up to 4 weeks.

Trial registration Clinicaltrials.gov Identifier: UMIN000043084. Registered 21/01/2021. https://center6.umin.ac.jp/cgi-open-bin/ctr_e/ctr_view.cgi?recptno=R000049188.

## Introduction

Nicotinamide adenine dinucleotide (NAD+) has long been known as a coenzyme for various redox reactions in major energy-generating pathways such as glycolysis, TCA circuitry, and beta-oxidation^1,2^. In addition, NAD+ is consumed as a substrate for the sirtuin family (SIRT1-7) of NAD+-dependent deacetylases/deacylases, poly ADP-ribose polymerase (PARP), and cluster of differentiation 38 (CD38), which catalyzes the synthesis and hydrolysis of cyclic ADP-ribose (cADPR). Thus, NAD+ is involved in various life phenomena such as DNA repair, post-translational modifications of proteins, epigenetic gene regulatory mechanisms, and circadian rhythms^3–8^.

Furthermore, recent studies have shown that eukaryotes have decreased NAD+ levels with aging in limited tissues or cells. With respect to mice, age-related reductions in NAD+ levels have been shown in skeletal muscles and some adipose tissues, as well as in the hippocampal region of the brain^9^. Decreased intracellular NAD+ levels affect the activity of SIRT1 and enzymes in the NAD+ salvage pathway, which require NAD+ as a cofactor, and is strongly implicated in Cockayne syndrome^10^. *Nampt* deletion is also strongly associated with reduced muscle strength and endurance, as it alters Ca^2+^ homeostasis and reduces mitochondrial function^11,12^_._ Therefore, preventing a decrease in intracellular NAD^+^ levels may help to prevent and treat these diseases^13,14^. Because NAD+ is impermeable to cell membranes, it is thought that the direct administration of NAD+ does not efficiently increase intracellular NAD+ levels^15^, and studies have therefore attempted to improve the intracellular NAD+ level uptake by administering precursors (NMN and NR) of the NAD+ biosynthetic pathway^16^.

Four different mammalian NAD+ biosynthesis metabolic pathways have been identified. The first is the de-novo pathway originating from L-tryptophan; the second is the Preiss-Handler pathway originating from nicotinic acid (NA), and the third is the salvage pathway, which starts from nicotinamide (NAM) and synthesizing NAD+ through a two-step enzymatic reaction. NAM is converted to NMN. NMN is subsequently biosynthesized to NAD+ by nicotinamide mononucleotide adenylyltransferase (NMNAT) in the second enzymatic step^1,2,17^. The fourth is the conversion pathway from nicotinamide riboside (NR), the nucleoside form of NMN, which is converted to NMN by nicotinamide riboside kinase (NRK1 and NRK2), followed by the biosynthesis of NAD+ by NMNAT^17^.

Recently, Grozio et al. confirmed that Slc12a8, which is highly expressed in the small intestine of mice, intracellularly transports NMN^18^. This suggests that Slc12a8 may be a transporter protein of NMN. On the other hand, the NAD+ metabolic flux analysis by Liu et al. that used NAD+ precursors containing stable isotopes showed that NMN and NR administered to mice were not directly metabolized to NAD+ but to NAM before reaching the obliterating tissues in the body^19^. In addition, it has also been reported that orally administered NAM was converted to NA by the gut microbiota and absorbed from the colon as NA ^20^. Yaku et al. showed that orally administered NR was converted to NAM by BST1 and then to NA by the microbiota ^21^. These studies suggest that orally administered uptake of NAD+ precursors and NAD+ biosynthesis involve various metabolic steps and microflora.

Moreover, several studies have reported the efficacy of NMN administration in increasing NAD+ levels in certain cell or tissue in various diseases and disorders, including age-related diseases^22^, obesity-related metabolic disorders^23,24^, diabetic nephropathy^25^, ischemic reperfusion injury^26,27^, improving cognitive function and depression-like behaviors^28–30^, improving reproductive function^31,32^, immunostimulation^33^, and hematopoietic effects^34^. Similarly, an intracellular NAD+ precursor of NR, has also been investigated in numerous studies, and Sun et al. found that NR supplementation improved NAD+ homeostasis in dyskeratosis congenita (DC) cells, a disorder of telomere maintenance, improved the effect of NR on DC cells, and ameliorated the cellular consequences of telomere dysfunction in DC cells^35^. NR supplementation also prevented weight gain in mice fed high-fat diet, as well as increased insulin sensitivity and mitochondrial mass in skeletal muscle^36^. Furthermore, Damgaard et al. showed that intravenous injection of NR increased NAD+ in mouse skeletal muscle without affecting respiratory capacity or insulin sensitivity^37^. The administration of NMN and NR to rodents, *Caenorhabditis elegans* (nematodes), and *Drosophila melanogaster* (fruit flies) has been shown to prolong lifespan and alleviate age-related physiological decline^38–40^. In other reports, NMN and NR have been suggested to bind to ACE2 and IMPDH, along with SARS-CoV-2 viral proteins (Spro, Mpro, PLpro, RdRp) by molecular docking and dynamics simulations, suggesting a potential therapeutic effect of NMN and NR in the treatment of COVID-19^41^.

Since various beneficial bioactivities of NMN have been reported in both *in-vivo* and *in-vitro* studies, several human clinical trials have been performed in recent years. A first study evaluated the safety of single oral doses of 100, 250, and 500 mg of NMN in healthy men, and reported that oral administration of NMN did not produce values exceeding normal physiological fluctuations in hematological and clinical biochemical tests^42^. A second study examined the efficacy of 250 mg/day of NMN, orally administered for 10 weeks, in improving glucose metabolism in overweight or obese postmenopausal women with prediabetes. The authors reported a significant improvement (an average of 25%) in insulin sensitivity and glucose uptake, which was reduced in type 2 diabetes and pre-diabetic patients^43^. A third study investigated the efficacy of oral administration of low-dose (150 mg twice daily), medium-dose (300 mg twice daily), and high-dose (600 mg twice daily) NMN on aerobic exercise capacity in healthy male and female amateur runners for 6 weeks. This study reported a dose-dependent increase in skeletal muscle oxygen utilization and improvement in aerobic capacity during exercise training^44^. In these studies, none of the human clinical trials reported any significant changes in body composition or serious adverse events during the study period^42–44^.

However, previous clinical studies with NMN have not reported on the safety of repeated daily oral administration of ≥ 1000 mg/shot^42–44^. In recent years, NMN supplements have become available on the market and are being ingested by consumers worldwide as healthcare products. In general, the commercially available NMN content ranges from 50 to 150 mg/capsule, but some consumers overdose on multiple capsules at a time. Moreover, there is a lack of evidence from human clinical trials and preclinical studies to support the safety of NMN used in these products. Therefore, we performed a randomized, double-blind, placebo-controlled, parallel-group study to evaluate the safety of 1250 mg of NMN when administered once daily for up to 4 weeks in healthy adult men and women.

## Methods

### Test compound

β-Nicotinamide mononucleotide (NMN) was manufactured by Mitsubishi Corporation Life Sciences Limited (Tokyo, Japan). The NMN used in this human clinical trial is H^+^ crystalline form (C_11_H_16_N_2_O_8_P).

### Composition and nutritional composition of the study foods and dose method

The test foods were provided to the subjects as a packaged powder. The composition of the test foods is shown in Table 1. The nutritional composition of the test foods is shown in Table 2. The test foods were completely dissolved in water (200 ml) and consumed once a day.

**Table 1:** Composition of the test foods.

Component	Placebo	NMM
	Single dose (g)	
NMN	–	1.25
Maltitol	3.75	3.75
Acesulfame potassium	0.03	0.03
Dextrin	5.00	5.00
Citric acid anhydrous	0.50	0.50
Flavor	0.10	0.10
Total amount	9.38	10.63

**Table 2:** Nutrition content of the test foods.

Component	Placebo	NMN
	Per serving (g)	
Moisture	0.35	0.43
Ash	0.00	0.29
Protein	0.00	0.51
Fat	0.00	0.00
Carbohydrate	9.03	9.40

### Bacterial reverse mutation test (Ames test)

The strains used for the Ames test were *Salmonella typhimurium* (TA100, TA1535, TA98, and TA1537) and *Escherichia coli* (WP2*uvrA*). Screening for the presence of NMN-induced revertant mutations was performed by preincubating each strain with and without S9Mix, and assessing metabolic activity. NMN concentrations were evaluated at 313, 625, 1250, 2500, and 5000 µg/plate. The Ames study was conducted by the Bozo Research Center, Inc. (Tokyo, Japan).

### Design of the human clinical study

**Figure 1:**
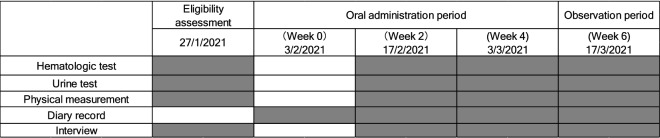
Study design.

The number of subjects in this study was set based on the number of subjects in previous NMN clinical trials^42^. A randomized, double-blind, placebo-controlled, parallel-group study was conducted on 31 healthy adult men and women aged 20–65 years who met the eligibility criteria. Examinations (anthropometry, hematology, clinical biochemistry, and urinalysis) and questionnaires were administered before intake (February 3, 2021), and in week 2 (February 17, 2021), week 4 (March 3, 2021), and after the observation period (March 17, 2021) (Fig. 1). During the intervention period, daily records were kept regarding confirmed intake of the test foods, menstruation, physical condition, dietary investigations, medications, and functional foods (e.g., supplements). This human study met the requirements of CONSORT 2010 and adhered to the ethical principles of the Declaration of Helsinki. All subjects were given a full explanation of the study (objectives, research methods, risks, and privacy concerns), and all subjects provided signed informed consent. The study was approved by the Clinical and Ethical Review Committee of Yoga Allergy Clinic (4-32-16, Yoga, Setagaya-ku, Tokyo, Japan, jim@medipharma.co.jp) on January 15, 2021, and registered in UMIN-CTR (UMIN000043084) before the start of the study (January 21, 2021). This study was conducted at Pharma Foods International Co., Ltd. (Kyoto, Japan).

### Exclusion criteria and randomization of subjects

Exclusion criteria were as follows:

1. A medical history of malignant tumor, heart failure, or myocardial infarction.
2. Currently undergoing treatment for any of the following chronic diseases: atrial fibrillation, arrhythmia, hepatic disorder, renal disorder, cerebrovascular disorder, rheumatism, diabetes, dyslipidemia, hypertension, and other chronic diseases.
3. Subjects who are currently taking medications (including herbal medicines).
4. Subjects who are allergic to medications and the test food-related products.
5. Subjects who are pregnant, breastfeeding, or planning to become pregnant.
6. Subjects who are judged as ineligible to participate in the study by the physician.

After application of the above exclusion criteria, eligible healthy men and women between the ages of 20 and 65 years were admitted to the study based on the results of hematological tests (triglycerides, LDL cholesterol, fasting blood glucose, HbA1c, AST (GOT), ALT (GPT), γ-GT, serum amylase, creatinine, and uric acid levels) that were judged by the physician to be acceptable for participation in the study. Subjects were randomly assigned to two groups, placebo and NMN, using a random number table by the controller of Pharma Foods International Co., Ltd., who was not involved in the study. The controller sealed the allocation sheets and kept them in a sealed envelope until the data analysis was completed.

### Instructions to study participants

1. Consume the test food according to the prescribed dosage and administration.
2. Avoid excessive drinking and eating from 1 week before the study until the end, and do not change diet or lifestyle.
3. Do not start taking any new supplements other than the test food during the study period.
4. Do not drink alcohol or exercise excessively the day before the test.
5. Do not eat or drink for 6 h prior to blood collection. Subjects may drink water but no functional drinks.
6. In case of any change in physical condition during the test period, the test management organization was immediately contacted. Subsequent actions should be taken according to instructions from the test management organization.

### Medical treatment of subjects during the study

Spot medications for minor symptoms, such as colds and headaches, were allowed. When medication was taken, it was noted in the medication confirmation column of the diary, and the reason for taking the medication was communicated to the study group.

### Primary outcomes

Primary outcomes in this study set hematological tests, clinical biochemical tests, body composition and vital signs, urinalysis, and adverse events.

### Adverse events

In this study, an adverse event was defined as a "new onset of abnormality" or "exacerbation" during the study period. In the event of an adverse event, the principal investigator was taken necessary and appropriate measures immediately and decided whether the participant could continue the study. The principal investigator evaluated the causal relationship between adverse events and test food. The evaluation results for adverse events were reported in writing.

### Statistical analysis

Statistical analyses were performed using SPSS Statistics V25 (IBM) or the Microsoft Excel 2016. Two-way analysis of variance was used to determine statistical significance for safety evaluation in this study, and Dunnett's post-hoc test (two-tailed test) was used to compare the initial value measured at week 0 with all subsequent measurements. Between-group comparisons were made using an unpaired t-test (two-tailed test) or Welch's t-test (white blood cell count and AST, ALT, γ-GT, arterial stiffness index, blood glucose, urea nitrogen, serum iron, total ketones, acetoacetic acid, and 3-hydroxybutyric acid only), and measurements were compared at weeks 0, 2, and 4 of intake, and the observation period (week 6). All data are expressed as mean ± SD, and the significance level of within-group comparisons was set at *P < 0.05, or **P < 0.01 vs week 0. The significance level of between-group comparisons was set at #*P* < 0.05, or ##*P* < 0.01 vs placebo. In addition, subjects who consumed less than 80% of the test food and did not comply with the study instructions were excluded from the statistical analysis.

## Results

### Bacterial reverse mutation test (Ames test)

The results of the Ames test are presented in Supplementary Table S1. The number of revertant mutant colonies treated with NMN did not increase more than two-fold compared to the number of negative controls in any of the strains, regardless of the presence or absence of metabolic activity. There was no increase in revertant mutant colonies at any of the treatment concentrations (313, 625, 1250, 2500, and 5000 µg/plate).

### Screening of subjects and basic data

**Figure 2:**
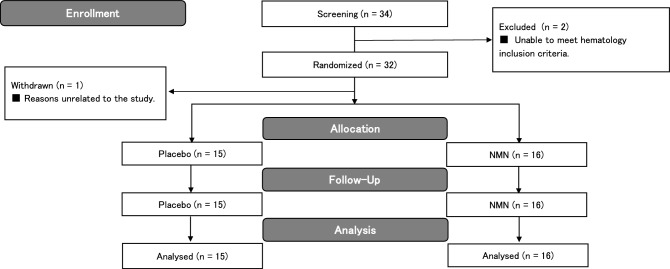
Flow diagram illustrating the phases of this randomized, double-blind, placebo-controlled, parallel-group study.

The subjects for this study were fully informed about the study's purpose, methods, and contents, and written informed consent for participation was obtained from 34 subjects. Pre-study screening tests were performed on January 27, 2021, and two subjects were excluded because they did not meet the criteria based on their hematological laboratory test results. After screening, 32 healthy men and women between 20 and 65 years of age were selected as subjects. The male-to-female ratio was 14 men and 18 women. The selected subjects were randomly assigned to the placebo group or the NMN group by a staff member who was not involved in the study, using a random number table with the following allocation factors: sex ratio, age, body weight, body fat percentage, BMI, and blood pressure. The allocation table was sealed and was only opened after data analysis was completed. Since one subject withdrew for reasons unrelated to the study before the start of the study, the final number of subjects was 31 (Fig. 2). Fifteen subjects were included in the placebo group, with a male-to-female ratio of seven males and eight females, while sixteen subjects were included in the NMN group, with a male-to-female ratio of seven males and nine females. The mean age, body weight, body fat percentage, BMI, systolic blood pressure, and diastolic blood pressure measurements of both groups are listed in Table 3. In this study, there was no heterogeneity in the physical data of the subjects in either group (Table 3). All 31 subjects complied with the study instructions throughout the intervention period, and their data were thus included in the subsequent analyses (Fig. 2). During the study period, the mean consumption rate of the test foods was 98.3 ± 4.0% in the placebo group and 98.9 ± 2.5% in the NMN group (Table 3).

**Table 3:** Subject characteristics and intake ratio.

Test items	Placebo (n = 15)	NMN (n = 16)
	Mean ± S.D.	
Age (years)	35.7 ± 7.2	35.1 ± 7.0
Male (n)	7	7
Female (n)	8	9
Weight (kg)	61.0 ± 12.2	61.9 ± 9.9
Body fat percentage (%)	25.1 ± 6.4	26.7 ± 7.1
BMI (kg/m^2^)	22.1 ± 3.3	22.9 ± 2.7
Systolic blood pressure (mmHG)	128.8 ± 14.9	127.2 ± 14.9
Diastolic blood pressure (mmHG)	80.8 ± 11.1	80.4 ± 11.9
Heart rate (bpm)	72.1 ± 11.0	77.4 ± 12.8
Intake ratio (%)	98.3 ± 4.0	98.9 ± 2.5

*BMI* body mass index, *S.D.* standard deviation.

### Hematological tests

The results of the hematological tests are shown in Table 4. All measurements in the NMN and Placebo groups were within the clinical laboratory reference values, and there were no significant differences within or between groups.

**Table 4:** Hematologic tests performed during the oral administration period.

Test items	Clinical laboratory reference values	Group	Week 0	Week 2	Week 4	Week 6 (observation period)
			Mean ± S.D.	Mean ± S.D.	Mean ± S.D.	Mean ± S.D.
White blood cell count (× 10^3^/μL)	Female: 3.6–9.0	Placebo	5.8 ± 0.8	5.9 ± 1.0	5.5 ± 0.8	5.7 ± 1.1
	Male: 3.9–9.8	NMN	5.9 ± 1.7	5.1 ± 1.3	5.3 ± 1.5	5.8 ± 1.8
Red blood cell count (× 10^4^/μL)	Female: 370–500	Placebo	473.1 ± 38.0	478.0 ± 34.9	464.9 ± 37.2	456.3 ± 39.3
	Male: 430–570	NMN	489.8 ± 40.3	487.4 ± 43.4	479.6 ± 43.3	466.8 ± 39.9
Hemoglobin (g/dL)	Female: 11.3–15.2	Placebo	14.8 ± 1.3	15.0 ± 1.3	14.7 ± 1.5	14.4 ± 1.5
	Male: 13.5–17.6	NMN	14.9 ± 1.2	14.9 ± 1.4	14.8 ± 1.5	14.5 ± 1.2
Hematocrit (%)	Female: 34–45	Placebo	44.5 ± 3.7	44.8 ± 3.7	43.6 ± 3.9	42.6 ± 3.8
	Male: 40–52	NMN	44.9 ± 3.7	44.8 ± 4.0	44.0 ± 4.1	42.7 ± 3.5
Platelet count (× 10^4^/μL)	Female: 13.0–36.9	Placebo	25.3 ± 5.2	25.1 ± 5.0	24.1 ± 5.5	24.2 ± 5.7
	Male: 13.1–36.2	NMN	26.5 ± 3.4	27.2 ± 4.1	25.6 ± 4.4	26.2 ± 3.8
MCV (fl)	Female: 79–100	Placebo	94.0 ± 4.3	93.7 ± 3.9	93.8 ± 3.8	93.3 ± 3.6
	Male: 83–102	NMN	91.9 ± 4.8	91.9 ± 4.4	91.8 ± 4.8	91.7 ± 4.6
MCH (pg)	Female: 26.3–34.3	Placebo	31.3 ± 1.5	31.4 ± 1.6	31.6 ± 1.6	31.6 ± 1.8
	Male: 28.0–34.6	NMN	30.5 ± 1.8	30.6 ± 1.8	30.9 ± 1.9	31.1 ± 2.0
MCHC (%)	Female: 30.7–36.6	Placebo	33.3 ± 0.5	33.5 ± 0.5	33.7 ± 0.7	33.8 ± 0.9
	Male: 31.6–36.6	NMN	33.2 ± 0.8	33.2 ± 0.8	33.6 ± 1.1	33.9 ± 1.1
HbA1c (NGSP) (%)	4.6–6.2	Placebo	5.3 ± 0.2	5.2 ± 0.2	5.3 ± 0.2	5.2 ± 0.3
		NMN	5.3 ± 0.2	5.2 ± 0.2	5.3 ± 0.2	5.2 ± 0.2

*MCV* mean corpuscular volume, *MCH* mean corpuscular hemoglobin concentration, *MCHC* mean corpuscular hemoglobin concentration, *NGSP* national glycohemoglobin standardization program.

The number of participants in the placebo group was n = 15, while the NMN group comprised n = 16. Statistical significance was determined by two-way analysis of variance and Dunnett's post hoc-test (two-tailed test) was used to compare the initial value measured at week 0 with all subsequent measurements. Between-group differences comparisons were made using a paired t-test or Welch’s t-test (only white blood cell count).

### Clinical biochemical tests

The results of the clinical biochemical tests are shown in Table 5. Only the sodium placebo group showed a significant decrease in the count at week 2 (*p* = 0.0148) compared to week 0. In the NMN group, LD (LDH) levels at week 0 and total protein during the observation period were significantly higher between groups compared to the placebo group (*p* = 0.0340 and 0.0310, respectively). In contrast, the A/G ratio and sodium levels in the NMN group at week 0 were significantly lower than those in the placebo group (*p* = 0.0345 and 0.0283, respectively). However, all of the clinical biochemical test items that showed significant differences between the groups in this study were within the clinical laboratory reference values.

**Table 5:** Clinical biochemical tests performed during the oral administration period.

Test items	Clinical laboratory reference values	Group	Week 0	Week 2	Week 4	Week 6 (observation period)
			Mean ± S.D.	Mean ± S.D.	Mean ± S.D.	Mean ± S.D.
**Enzymatic activity**						
AST (GOT) (U/L)	10–40	Placebo	20.1 ± 5.7	20.7 ± 5.4	20.7 ± 6.3	20.5 ± 7.0
		NMN	20.8 ± 7.2	23.6 ± 15.6	19.9 ± 7.7	22.3 ± 11.6
ALT (GPT) (U/L)	5–45	Placebo	20.5 ± 13.7	23.3 ± 14.2	21.9 ± 14.4	21.8 ± 15.9
		NMN	23.8 ± 17.3	28.8 ± 33.1	22.0 ± 19.2	25.6 ± 25.0
ALP (U/L)	110–340	Placebo	175.8 ± 41.7	177.5 ± 36.0	175.3 ± 40.4	173.5 ± 50.5
		NMN	197.3 ± 51.7	203.9 ± 57.0	198.9 ± 56.8	193.9 ± 58.2
γ-GT (U/L)	Female: 10–40	Placebo	32.7 ± 26.0	33.3 ± 25.7	33.9 ± 29.1	36.2 ± 33.2
	Male: 12–87	NMN	43.4 ± 64.4	44.9 ± 77.2	42.3 ± 71.3	46.0 ± 76.5
LD (LDH) (U/L)	107–230	Placebo	156.9 ± 16.8	158.7 ± 21.1	158.3 ± 22.0	161.7 ± 19.3
		NMN	174.8 ± 26.4 #	174.0 ± 23.7	173.4 ± 28.6	174.0 ± 25.9
**Lipid metabolism**						
Triglyceride (mg/dL)	40–149	Placebo	77.5 ± 47.0	74.7 ± 35.9	77.7 ± 45.6	103.3 ± 104.7
		NMN	97.9 ± 57.8	84.8 ± 46.8	82.5 ± 55.7	99.3 ± 65.2
Total Cholesterol (mg/dL)	130–220	Placebo	209.3 ± 28.9	209.0 ± 24.9	207.1 ± 33.3	203.7 ± 30.7
		NMN	209.1 ± 38.6	218.6 ± 35.7	212.9 ± 38.0	210.8 ± 40.1
Free fatty acid (μEq/L)	150–600	Placebo	529.2 ± 290.3	409.3 ± 155.2	396.2 ± 209.5	338.8 ± 139.9
		NMN	414.1 ± 178.9	390.5 ± 196.2	405.9 ± 184.8	429.9 ± 194.9
HDL Cholesterol (mg/dL)	Female: 40–86	Placebo	68.6 ± 19.2	67.7 ± 16.2	64.5 ± 13.4	64.9 ± 11.0
	Male: 40–80	NMN	66.3 ± 13.6	67.3 ± 13.8	63.4 ± 12.5	65.3 ± 12.8
LDL Cholesterol (mg/dL)	70–139	Placebo	121.9 ± 24.8	121.1 ± 26.3	125.9 ± 34.4	117.1 ± 30.8
		NMN	122.3 ± 34.8	128.2 ± 36.6	130.9 ± 37.5	122.9 ± 35.9
LDL/HDL ratio	–	Placebo	1.9 ± 0.6	1.9 ± 0.6	2.1 ± 0.8	1.9 ± 0.6
		NMN	2.0 ± 0.9	2.0 ± 0.9	2.2 ± 0.9	2.0 ± 0.8
Lipoprotein (a) (mg/dL)	≤ 30	Placebo	22.8 ± 27.2	21.7 ± 25.4	21.3 ± 24.7	21.3 ± 26.0
		NMN	17.9 ± 16.8	16.3 ± 15.5	18.4 ± 17.1	17.6 ± 17.3
Arteriosclerosis index	≤ 4.5	Placebo	2.2 ± 0.7	2.2 ± 0.7	2.3 ± 0.8	2.2 ± 0.6
		NMN	2.3 ± 1.0	2.4 ± 1.0	2.5 ± 1.0	2.4 ± 1.0
**Carbohydrate metabolism**						
Blood Glucose (mg/dL)	70–109	Placebo	93.5 ± 8.8	90.4 ± 9.0	92.2 ± 6.8	90.8 ± 9.4
		NMN	91.3 ± 4.1	90.1 ± 5.6	92.9 ± 5.9	91.8 ± 4.7
Insulin (μU/mL)	2–11	Placebo	6.9 ± 2.9	7.1 ± 3.1	5.8 ± 2.8	6.0 ± 3.2
		NMN	7.1 ± 3.8	7.9 ± 4.8	7.4 ± 3.8	6.3 ± 2.8
**Nitrogen compound**						
Urea Nitrogen (mg/dL)	8.0–20.0	Placebo	12.2 ± 2.5	13.0 ± 2.6	12.8 ± 3.1	12.7 ± 2.9
		NMN	12.6 ± 3.4	12.5 ± 5.0	12.9 ± 3.5	12.8 ± 4.5
Uric acid (mg/dL)	Female: 2.5–7.0	Placebo	4.6 ± 1.2	4.9 ± 1.1	5.0 ± 1.3	4.9 ± 1.3
	Male: 3.6–7.0	NMN	5.0 ± 1.4	5.1 ± 1.4	5.1 ± 1.5	5.2 ± 1.4
Creatinine (mg/dL)	Female: 0.47–0.79	Placebo	0.76 ± 0.16	0.76 ± 0.15	0.75 ± 0.16	0.74 ± 0.16
	Male: 0.61–1.04	NMN	0.78 ± 0.13	0.75 ± 0.11	0.73 ± 0.12	0.74 ± 0.14
**Proteins**						
Total Protein (g/dL)	6.5–8.3	Placebo	7.5 ± 0.3	7.6 ± 0.2	7.5 ± 0.3	7.4 ± 0.2
		NMN	7.7 ± 0.3	7.7 ± 0.3	7.6 ± 0.3	7.6 ± 0.2 #
Albumin (g/dL)	3.8–5.2	Placebo	4.9 ± 0.3	4.9 ± 0.2	4.8 ± 0.3	4.7 ± 0.2
		NMN	4.8 ± 0.2	4.8 ± 0.3	4.8 ± 0.3	4.7 ± 0.3
A/G ratio	1.3–2.0	Placebo	1.9 ± 0.2	1.9 ± 0.3	1.8 ± 0.2	1.8 ± 0.2
		NMN	1.7 ± 0.2 #	1.7 ± 0.2	1.7 ± 0.2	1.7 ± 0.2
**Electrolytes**						
Sodium (mEq/L)	135–147	Placebo	141.8 ± 1.1	140.3 ± 1.8 *	141.6 ± 1.1	140.8 ± 1.4
		NMN	140.9 ± 0.9 #	140.8 ± 1.0	141.3 ± 1.5	140.6 ± 0.9
Potassium (mEq/L)	3.3–5.0	Placebo	4.4 ± 0.4	4.3 ± 0.3	4.1 ± 0.3	4.2 ± 0.3
		NMN	4.4 ± 0.5	4.4 ± 0.5	4.1 ± 0.3	4.3 ± 0.4
Calcium (mg/dL)	8.6–10.1	Placebo	9.2 ± 0.3	9.0 ± 0.2	9.0 ± 0.3	9.1 ± 0.4
		NMN	9.2 ± 0.3	9.1 ± 0.3	9.1 ± 0.2	9.1 ± 0.3
Chloride (mEq/L)	98–108	Placebo	101.9 ± 1.5	101.7 ± 1.8	101.1 ± 1.7	101.8 ± 2.1
		NMN	101.5 ± 1.7	102.3 ± 2.1	101.7 ± 2.1	101.8 ± 1.7
Inorganic Phosphorus (mg/dL)	2.5–4.5	Placebo	3.1 ± 0.4	3.3 ± 0.5	3.2 ± 0.5	3.2 ± 0.4
		NMN	3.2 ± 0.4	3.2 ± 0.4	3.2 ± 0.4	3.2 ± 0.4
**Metals**						
Magnesium (mg/dL)	1.8–2.5	Placebo	2.3 ± 0.1	2.3 ± 0.1	2.3 ± 0.2	2.2 ± 0.1
		NMN	2.3 ± 0.1	2.3 ± 0.1	2.3 ± 0.1	2.3 ± 0.1
Serum Iron (μg/dL)	Female: 40–175	Placebo	108.1 ± 27.5	111.5 ± 33.7	92.9 ± 26.8	102.8 ± 44.9
	Male: 55–185	NMN	113.1 ± 45.5	114.8 ± 48.1	107.2 ± 49.9	110.6 ± 37.3
**Ketone bodies carboxylic acid**						
Total Ketone bodies (μmol/L)	≤ 130	Placebo	89.9 ± 95.7	70.4 ± 54.3	85.9 ± 100.6	48.5 ± 38.9
		NMN	58.5 ± 57.5	65.3 ± 65.6	72.8 ± 72.5	68.1 ± 89.8
Acetoacetic acid (μmol/L)	≤ 55	Placebo	17.2 ± 17.4	14.7 ± 9.9	17.7 ± 21.6	9.7 ± 7.3
		NMN	12.6 ± 10.3	15.2 ± 14.9	14.5 ± 14.5	13.6 ± 16.3
3-Hydroxybutyric acid (μmol/L)	≤ 85	Placebo	72.7 ± 78.7	55.7 ± 45.4	68.2 ± 79.3	38.8 ± 32.1
		NMN	45.9 ± 47.5	50.1 ± 50.9	58.3 ± 58.2	54.6 ± 73.6

*AST* aspartate aminotransferase, *GOT* glutamate oxaloacetate transaminase, *ALT* alanine aminotransferase, *GPT* glutamic pyruvic transaminase, *ALP* alkaline phosphatase, *γ-GT* gamma-glutamyltransferase, *LD (LDH)* lactate dehydrogenase, *HDL* high-density lipoprotein, *LDL* low-density lipoprotein.

The number of participants in the placebo group was n = 15, while the NMN group comprised n = 16. Statistical significance was determined by two-way analysis of variance and Dunnett's post-hoc test (two-tailed test) was used to compare the initial value measured at week 0 with all subsequent measurements. Between-group difference comparisons were made using a paired t-test or Welch’s t-test (only AST and ALT, γ-GT, arteriosclerosis index, blood glucose, urea nitrogen, serum iron, total ketone bodies, acetoacetic acid, 3-hydroxybutyric acid). Statistically significant within-group differences were defined as *p < 0.05 versus Week 0. Statistically significant between-group differences were defined as #*p* < 0.05 versus placebo.

### Body composition and vital signs

Body composition and vital signs data are shown in Supplementary Table S2. All measurements in the NMN and Placebo groups were within the clinical laboratory reference values, and there were no significant differences within or between groups.

### Urinalysis

Urinalysis results are shown in Supplementary Tables S3 and S4. With respect to urinary protein (week 0 and week 4, observation period) and urobilinogen (week 0) levels, the results of some subjects were outside of the clinical laboratory reference values in both the placebo and the NMN groups. For urobilinogen (week 2), urine occult blood (week 2 and observation period), urine glucose (week 0), and urine ketone bodies (weeks 2 and 4), there were subjects whose results were outside of the clinical laboratory reference values only in the placebo group. The urine bilirubin results for both groups were all within the clinical laboratory reference values. There was no significant difference in urine specific gravity and urine pH within or between groups during the study.

### Adverse events

Reports of adverse events during the study period are presented in Table 6. Five adverse events were observed during the study period, but the study principal investigator determined that there was no direct causal relationship between the administration of the test food and any of the adverse events.

**Table 6:** Adverse events during the clinical trial.

Group	Placebo	NMN	NMN	NMN	NMN
Subject No.	9	11	16	19	23
Adverse Event	Loose stool	Common cold	High blood pressure	Loose stool	Acne vulgaris
Occurrence of adverse events	Oral administration period	Oral administration period	Oral administration period	Oral administration period	Oral administration period
Extent of symptom	Mild	Moderate	Mild	Mild	Mild
Availability of treatment	Without	With	Without	Without	Without
Outcome	Recovery	Recovery	Remission	Recovery	Recovery
Causality	Unrelated	Unrelated	Unrelated	Unrelated	Unrelated
Reasons for determining causality	The causal relationship with this test food was irrelevant due to an incidental event	The causal relationship with this test food was irrelevant because of the infection	The causal relationship with this test food was irrelevant due to an incidental event	The causal relationship with this test food was irrelevant due to an incidental event	The causal relationship with this test food was irrelevant due to an incidental event
Resumption or discontinuation of the study	Resumption	Resumption	Resumption	Resumption	Resumption

## Discussion

In this study, we evaluated the safety of NMN intake in healthy adult men and women. Our findings reveal that oral administration of 1250 mg of NMN, when administered once daily for up to 4 weeks, was safe and well-tolerated in healthy adult men and women. The NMN manufactured by Mitsubishi Corporation Life Science Limited used in this study did not show mutagenicity in the Ames test. This result was identical to that reported by Cros et al.^45^, suggesting that NMN is a non-mutagenic compound.

A human clinical study on the safety of NMN was reported by Irie et al., who performed a single oral dose study of NMN (100 mg, 250 mg, and 500 mg) in healthy males and demonstrated the safety of single oral doses up to 500 mg^42^. In our study, no adverse physical effects were observed even after 4 weeks of repeated oral administration of 1250 mg NMN once a day, a high-dose compared to the single oral dose of 500 mg. Our study also assessed both healthy men and women. Our results indicate that oral administration of NMN did not have any adverse effects on the body in healthy males or females. In their report, Irie et al. and noted a significant increase in total serum bilirubin levels within clinical laboratory reference values. Irie et al. described a significant decrease in blood glucose and blood chloride in oral administration of NMN within the clinical laboratory reference values, but our study did not observe any significant decreases in these laboratory data. Blood creatinine is known to be an indicator of renal function, and in a subacute toxicity evaluation of NMN (1340 mg/day) in beagle dogs by You et al., a significant increase in blood creatinine levels was observed in the NMN group^46^, suggesting that oral administration of high-doses of NMN (1340 mg/day) has mild adverse effects on the kidneys in beagle dogs. However, in the report of Irie et al. (500 mg/day) and our current study (1250 mg/day), there was no significant increase in blood creatinine levels with oral administration of NMN^42^. Therefore, we consider that oral administration of NMN in humans has a low adverse effect on renal function.

The human efficacy studies by Yoshino et al. and Liao et al. also confirmed the supplemental safety assessments in postmenopausal women with prediabetes who were overweight or obese, with no reports of body composition changes or serious events^43,44^. Similarly, we did not observe any changes in body composition or serious adverse events during our study in healthy adult men and women. This suggests that NMN can be safely administered orally without altering body composition in healthy adults and patients with obesity and glucose metabolism diseases.

In recent years, oral administration of NR, which are intermediates for NAD+ biosynthesis similar to NMN, has been reported to increase blood NAD+ levels^47^. The safety of oral administration of NR in humans has also been evaluated. Conze et al. evaluated the kinetics, dose-dependence, and safety of oral intake of NR chloride (100, 300, and 1000 mg) in healthy overweight adult men and women^48^. They did not report any serious adverse events or facial flushing. Dollerup et al. tested the safety and potential for improvement of insulin sensitivity and other metabolic parameters in obese insulin-resistant men after administration of 2000 mg (1000 mg × 2/day) of high-dose NR for 12 weeks to test its safety and potential for improving insulin sensitivity and other metabolic parameters^49^. The validation results confirmed that no serious adverse events with NR supplementation, and blood tests were standard. Thus, similar to the results of these human clinical studies on the intake of NR above 1000 mg/day, no serious adverse events were observed for NMN intake ≥ 1000 mg/shot in this study.

Nicotinamide (NAM) and nicotinic acid (NA) are already available on the world market as dietary supplements. However, high oral doses of NAM and NA have been reported to be hepatotoxic to humans^50,51^, and an adverse effect of vasodilative flushing due to high NA intake has been shown^52^. The upper tolerable dose of NAM and NA for humans established by the European Commission and the UK Vitamin and Mineral Expert are 900 mg/day and 10 mg/day, respectively^53^. The present results indicate that NMN, the same NAD+ precursor as NAM and NA, can be administered orally to humans at doses 1250 mg once daily for up to 4 weeks without causing hepatotoxicity and vasodilative flushing, and is believed to have a higher upper tolerable limit compared to NAM and NA.

NAD+ precursors such as NMN are present in trace amounts in foods^39^. However, since they have been shown to undergo degradation by heating, it is unlikely that the body receives these through oral intake of cooked food^54^. Therefore, during oral administration throughout the intervention period, a direct effect on the study of NMN ingested from diets other than the test food can be expected to be negligible.

There were several limitations to our study. First, metabolomic analysis of NMN and its metabolites, such as NAD+, NAM, NR, N-methyl-2-pyridone-5-carboxamide (2Py), and N-methyl-4-pyridone-5-carboxamide (4Py), in the blood and urine samples was not performed during the study period. Metabolomic analysis can confirm the bioavailability of NMN and the disposition of NMN metabolites in the body when individuals are administered NMN and is expected to provide further data to support the safety of oral administration of NMN. Second, body composition, hematological, clinical biochemical, and urinalysis tests were used as criteria for safety in this study. In addition to these clinical laboratory tests, clinical physiological tests such as MRI, ECG, EEG, and, if possible, histological tests by biopsy should be performed to comprehensively verify the safety of NMN. Finally, our study was relatively small, and perform a more detailed analysis of NMN excessive intake, a larger number of subjects or a long-term intake safety study or cohort study is needed.

NMN is contained in natural foods such as edamame (immature soybeans), broccoli, avocados, tomatoes, and milk, but the amount consumed in the normal diet is likely to be less than 2 mg/day^16,39,43^. It is difficult to consume more than 250 mg/day of NMN in the normal diet, which is the intake established in previous NMN clinical trials^42–44^. Therefore, it is efficient to take NMN from dietary supplements containing a high NMN content. In recent years, the commercialization of NMN as a dietary supplement has been expanding on the global market, but evidence for the safety of NMN in humans has been limited. In this human clinical study, NMN manufactured by Mitsubishi Corporation Life Sciences Limited was shown to be safe and well-tolerated an oral dose of 1250 mg once daily for up to 4 weeks.

## Supplementary Information

Supplementary Table S1.

Supplementary Table S2.

Supplementary Table S3.

Supplementary Table S4.

## Data Availability

The datasets generated and analyzed during the current study are available by request.

## References

[1] Okabe K, Yaku K, Tobe K, Nakagawa T (2019). Implications of altered NAD metabolism in metabolic disorders. J. Biomed. Sci..

[2] Houtkooper RH, Cantó C, Wanders RJ, Auwerx J (2010). The secret life of NAD^+^: An old metabolite controlling new metabolic signaling pathways. Endocr. Rev..

[3] Katsyuba E, Auwerx J (2017). Modulating NAD^+^ metabolism, from bench to bedside. EMBO J..

[4] Imai S, Guarente L (2014). NAD^+^ and sirtuins in aging and disease. Trends Cell Biol..

[5] Navas LE, Carnero A (2021). NAD^+^ metabolism, stemness, the immune response, and cancer. Signal Transduct. Target. Ther..

[6] Nakahata Y, Bessho Y (2016). The circadian NAD^+^ metabolism: Impact on chromatin remodeling and aging. BioMed Res. Int..

[7] Nakahata Y, Sahar S, Astarita G, Kaluzova M, Sassone-Corsi P (2009). Circadian control of the NAD^+^ salvage pathway by CLOCK-SIRT1. Science.

[8] Levine DC (2020). NAD^+^ controls circadian reprogramming through PER2 nuclear translocation to counter aging. Mol. Cell..

[9] Peluso A, Damgaard MV, Mori M, Treebak JT (2021). Age-dependent decline of NAD^+^-universal truth or confounded consensus?. Nutrients.

[10] Scheibye-Knudsen M (2014). A high-fat diet and NAD(+) activate Sirt1 to rescue premature aging in Cockayne syndrome. Cell Metab..

[11] Frederick DW (2016). Loss of NAD homeostasis leads to progressive and reversible degeneration of skeletal muscle. Cell Metab..

[12] Basse AL (2021). *Nampt* controls skeletal muscle development by maintaining Ca^2+^ homeostasis and mitochondrial integrity. Mol. Metab..

[13] Ramja L, Chwalek K, Sinclair DA (2018). Therapeutic potential of NAD-boosting molecules: The *in vivo* evidence. Cell Metab..

[14] Xie N (2020). NAD^+^ metabolism: Pathophysiologic mechanisms and therapeutic potential. Signal Transduct. Target. Ther..

[15] Zocchi E (1999). Ligand-induced internalization of CD38 results in intracellular Ca^2+^ mobilization: Role of NAD^+^ transport across cell membranes. FASEB J..

[16] Yoshino J, Baur JA, Imai SI (2018). NAD(+) intermediates: The biology and therapeutic potential of NMN and NR. Cell Metab..

[17] Stein LR, Imai S (2012). The dynamic regulation of NAD metabolism in mitochondria. Trends Endocrinol. Metab..

[18] Grozio A (2019). Slc12a8 is a nicotinamide mononucleotide transporter. Nat. Metab..

[19] Liu L (2018). Quantitative analysis of NAD synthesis-breakdown fluxes. Cell Metab..

[20] Shats I (2020). Bacteria boost mammalian host NAD metabolism by engaging the deamidated biosynthesis pathway. Cell Metab..

[21] Yaku K (2021). BST1 regulates nicotinamide riboside metabolism via its glycohydrolase and base-exchange activities. Nat. Commun..

[22] Yoshino J, Mills KF, Yoon MJ, Imai S (2011). Nicotinamide mononucleotide, a key NAD(+) intermediate, treats the pathophysiology of diet- and age-induced diabetes in mice. Cell Metab..

[23] Uddin GM, Youngson NA, Sinclair DA, Morris MJ (2016). Head to head comparison of short-term treatment with the NAD(+) precursor nicotinamide mononucleotide (NMN) and 6 weeks of exercise in obese female mice. Front. Pharmacol..

[24] Uddin GM (2020). Administration of nicotinamide mononucleotide (NMN) reduces metabolic impairment in male mouse offspring from obese mothers. Cells.

[25] Yasuda I (2021). Pre-emptive short-term nicotinamide mononucleotide treatment in a mouse model of diabetic nephropathy. J. Am. Soc. Nephrol..

[26] Park JH, Long A, Owens K, Kristian T (2016). Nicotinamide mononucleotide inhibits post-ischemic NAD(+) degradation and dramatically ameliorates brain damage following global cerebral ischemia. Neurobiol. Dis..

[27] Yamamoto T (2014). Nicotinamide mononucleotide, an intermediate of NAD^+^ synthesis, protects the heart from ischemia and reperfusion. PLoS ONE.

[28] Hosseini L (2019). Nicotinamide mononucleotide and melatonin alleviate aging-induced cognitive impairment via modulation of mitochondrial function and apoptosis in the prefrontal cortex and hippocampus. Neuroscience.

[29] Tarantini S (2019). Nicotinamide mononucleotide (NMN) supplementation rescues cerebromicrovascular endothelial function and neurovascular coupling responses and improves cognitive function in aged mice. Redox Biol..

[30] Xie X (2020). Nicotinamide mononucleotide ameliorates the depression-like behaviors and is associated with attenuating the disruption of mitochondrial bioenergetics in depressed mice. J. Affect. Disord..

[31] Miao Y, Cui Z, Gao Q, Rui R, Xiong B (2020). Nicotinamide mononucleotide supplementation reverses the declining quality of maternally aged oocytes. Cell Rep..

[32] Bertoldo MJ (2020). NAD^+^ repletion rescues female fertility during reproductive aging. Cell Rep..

[33] Takeda K, Okumura K (2021). Nicotinamide mononucleotide augments the cytotoxic activity of natural killer cells in young and elderly mice. Biomed. Res..

[34] Vannini N (2019). The NAD-booster nicotinamide riboside potently stimulates hematopoiesis through increased mitochondrial clearance. Cell Stem Cell.

[35] Sun C (2020). Re-equilibration of imbalanced NAD metabolism ameliorates the impact of telomere dysfunction. EMBO J..

[36] Cantó C (2012). The NAD(+) precursor nicotinamide riboside enhances oxidative metabolism and protects against high-fat diet-induced obesity. Cell Metab..

[37] Damgaard MV (2022). Intravenous nicotinamide riboside elevates mouse skeletal muscle NAD+ without impacting respiratory capacity or insulin sensitivity. iScience.

[38] Zhang H (2016). NAD^+^ repletion improves mitochondrial and stem cell function and enhances life span in mice. Science.

[39] Mills KF (2016). Long-term administration of nicotinamide mononucleotide mitigates age-associated physiological decline in mice. Cell Metab..

[40] Fang EF (2019). NAD+ augmentation restores mitophagy and limits accelerated aging in Werner syndrome. Nat. Commun..

[41] Esam Z, Akhavan M, Lotfi M, Bekhradnia A (2022). Molecular docking and dynamics studies of nicotinamide Riboside as a potential multi-target nutraceutical against SARS-CoV-2 entry, replication, and transcription: A new insight. J. Mol. Struct..

[42] Irie J (2020). Effect of oral administration of nicotinamide mononucleotide on clinical parameters and nicotinamide metabolite levels in healthy Japanese men. Endocr. J..

[43] Yoshino M (2021). Nicotinamide mononucleotide increases muscle insulin sensitivity in prediabetic women. Science.

[44] Liao B (2021). Nicotinamide mononucleotide supplementation enhances aerobic capacity in amateur runners: A randomized, double-blind study. J. Int. Soc. Sports Nutr..

[45] Cros C, Cannelle H, Laganier L, Grozio A, Canault M (2021). Safety evaluation after acute and sub-chronic oral administration of high purity nicotinamide mononucleotide (NMN-C®) in Sprague-Dawley rats. Food Chem. Toxicol..

[46] You Y (2020). Subacute toxicity study of nicotinamide mononucleotide via oral administration. Front. Pharmacol..

[47] Trammell S (2016). Nicotinamide riboside is uniquely and orally bioavailable in mice and humans. Nat. Commun..

[48] Conze D, Brenner C, Kruger CL (2019). Safety and metabolism of long-term administration of NIAGEN (nicotinamide riboside chloride) in a randomized, double-blind, placebo-controlled clinical trial of healthy overweight adults. Sci. Rep..

[49] Dollerup OL (2018). A randomized placebo-controlled clinical trial of nicotinamide riboside in obese men: Safety, insulin-sensitivity, and lipid-mobilizing effects. Am. J. Clin. Nutr..

[50] Ito TK (2021). A single oral supplementation of nicotinamide within the daily tolerable upper level increases blood NAD^+^ levels in healthy subjects. Transl. Med. Aging.

[51] Knip M (2000). Safety of high-dose nicotinamide: A review. Diabetologia.

[52] MacKay D, Hathcock J, Guarneri E (2012). Niacin: Chemical forms, bioavailability, and health effects. Nutr. Rev..

[53] Scientific Committee on Food. *Tolerable Upper Intake Levels for Vitamin S and Minerals* (2006). https://www.efsa.europa.eu/sites/default/files/efsa_rep/blobserver_assets/ndatolerableuil.pdf.

[54] Ummarino S (2017). Simultaneous quantitation of nicotinamide riboside, nicotinamide mononucleotide and nicotinamide adenine dinucleotide in milk by a novel enzyme-coupled assay. Food Chem..

