# Supplementary material for: Safety evaluation of β-nicotinamide mononucleotide oral administration in healthy adult men and women
Source: Sci Rep. 2022 Aug 24;12:14442. doi: 10.1038/s41598-022-18272-y (PMC9400576; doi:10.1038/s41598-022-18272-y)
Supplement: Supplementary file 1 — Supplementary Table S1. [file 41598_2022_18272_MOESM1_ESM.docx]

**Table S1. Colony number in bacterial reverse mutation test for NMN**

AF-2, 2-(2-furyl)-3-(5-nitro-2-furyl)acrylamide; SAZ, sodium azide; ICR-191, 2-methoxy-6-chloro-9-[3-(2-chloroethyl)aminopropylamino]acridine dihydrochloride; B[*α*]P, benzo[*α*]pyrene; 2AA, 2-aminoanthracene.
