# Supplementary material for: Safety evaluation of β-nicotinamide mononucleotide oral administration in healthy adult men and women
Source: Sci Rep. 2022 Aug 24;12:14442. doi: 10.1038/s41598-022-18272-y (PMC9400576; doi:10.1038/s41598-022-18272-y)
Supplement: Supplementary file 2 — Supplementary Table S2. [file 41598_2022_18272_MOESM2_ESM.docx]

**Table S2. Body composition and vital signs during the oral administration period**

The number of participants in the placebo group was n = 15, while the NMN group comprised n = 16. Statistical significance was determined by two-way analysis of variance and Dunnett's post hoc-test (two-tailed test) was used to compare the initial value measured at week 0 with all subsequent measurements. Between-group comparisons were made using an unpaired t-test (two-tailed test).
